# Five animal phyla in glacier ice reveal unprecedented biodiversity in New Zealand's Southern Alps

**DOI:** 10.1038/s41598-021-83256-3

**Published:** 2021-02-16

**Authors:** Daniel H. Shain, Philip M. Novis, Andrew G. Cridge, Krzysztof Zawierucha, Anthony J. Geneva, Peter K. Dearden

**Affiliations:** 1 Biology Department, Rutgers The State University of New Jersey, Camden, NJ 08103 USA; 2 Allan Herbarium, Manaaki Whenua-Landcare Research, Lincoln, 7608 New Zealand; 3 Genomics Aotearoa and Department of Biochemistry, University of Otago, Dunedin, 9054 New Zealand; 4 Department of Animal Taxonomy and Ecology, Adam Mickiewicz University in Poznań, 61-614 Poznań, Poland

**Keywords:** Evolution, Zoology

## Abstract

Glacier ice is an extreme environment in which most animals cannot survive. Here we report the colonization of high elevation, climate-threatened glaciers along New Zealand’s southwestern coast by species of Arthropoda, Nematoda, Platyhelminthes, Rotifera and Tardigrada. Based on DNA barcoding and haplotype-inferred evidence for deep genetic variability, at least 12 undescribed species are reported, some of which have persisted in this niche habitat throughout the Pleistocene. These findings identify not only an atypical biodiversity hotspot but also highlight the adaptive plasticity of microinvertebrate Animalia.

## Introduction

Glacier ecosystems are an inhospitable environment for most animals. The cumulative weight of overlying snow/ice compresses deep subsurface ice to densities > 900 kg/m^3^, effectively excluding physical space for even the smallest single-celled microbes^1, 2^. Prior to compression, however, upper layers of ice (i.e., weathered surface and several metres below) maintain ultrastructural spaces between crystal interfaces, forming arrays of microchannels that connect with the glacial surface^3, 4^. On maritime glaciers, those most threatened by our changing global climate^5–7^, ultrathin films of water fill these veinous aquifers and provide a microenvironment for extremophilic life. Permanently cold temperatures (0 °C and below), high UV radiation, nutrient-poor and hydrologically-limiting conditions constrain organismal diversity in this habitat to specialized psychrophilic taxa, predominantly single-celled microbes^8–11^.

Wright (1887) discovered the first glacially-obligate, multicellular animal—the glacier ice worm, *Mesenchytraeus solifugus* (phylum Annelida)–inhabiting Muir Glacier, Alaska^12, 13^, thereafter reported on glaciers throughout the Pacific Northwest^14, 15^. These worms inhabit glacier ice above the equilibrium line altitude (ELA), which separates snow accumulation and ablation zones, respectively. Ice worms also appear occasionally in meltwater pools common within the ablation zone (e.g., cryoconite holes), which support multiple trophic levels across domains of life including apex meiofauna^16–18^. More recently, two species of bdelloid Rotifera were discovered on maritime, Icelandic glaciers, identifying the second known animal phylum with representatives inhabiting glacier ice^19^.

Coastal glaciers in New Zealand’s Southern Alps are exceptional in that they descend steeply into native rainforest and experience particularly high levels of orographic precipitation^20–22^. Moreover, predominant oceanic westerlies channel wind up river valleys, leading to turbulent mixing of organic and inorganic debris^23, 24^. Significantly, glaciers in the Southern Alps advanced between 1983 and 2008 as a consequence of anthropogenic regional cooling^25^, but are now in rapid retreat comparable with glacial melting worldwide^5, 26, 27^.

The unusual climatology and geomorphology of the region, coupled with its proximity to rich sources of biodiversity in lower rainforests, prompted us to survey accessible glaciers in the region for animal life. We show here that taxa representing five animal phyla co-occur on Southern Alps, New Zealand glaciers, four of which (Arthropoda, Nematoda, Platyhelminthes, Tardigrada) are not reported previously in glacier ice.

## Results and discussion

**Figure 1:**
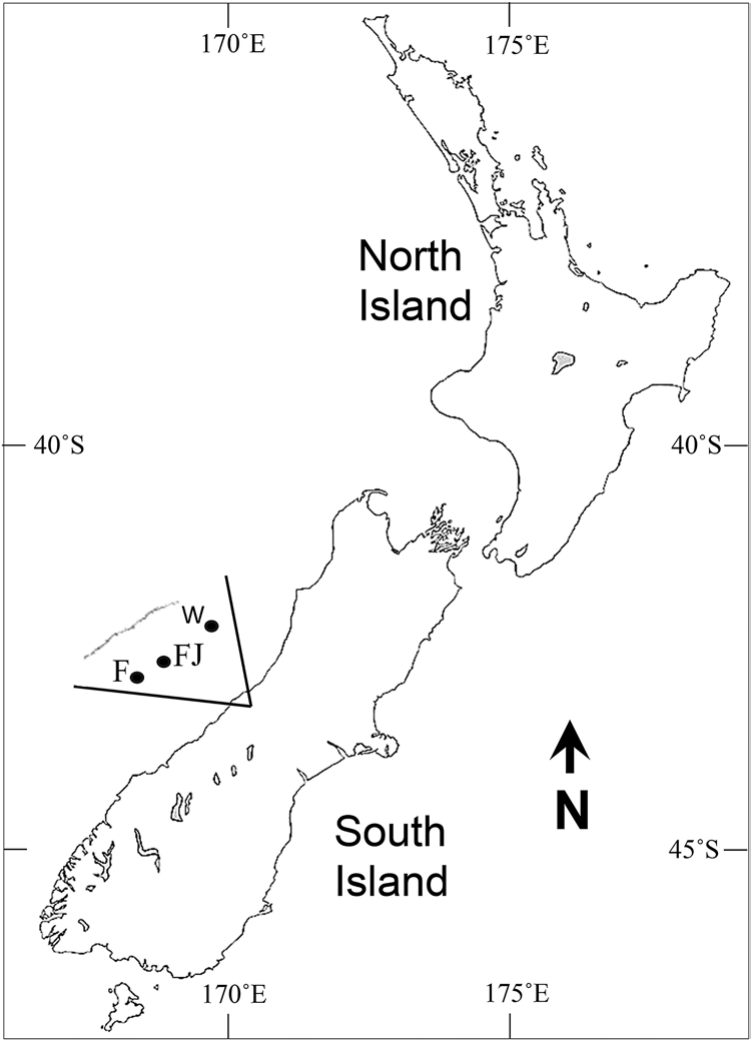
Localities of New Zealand glacier field sites. Collections were made on April 28, 2020, along a NE  SW transect spanning ~ 35 km, from Whataroa (W; − 43.4002, 170.5231; 4,859 ft) to Franz Joseph (FJ; − 43.6575, 170.2374; 6,890 ft) to Fox (F; − 43.5331, 170.1271; 6,483 ft) Glaciers. North is up.

**Figure 2:**
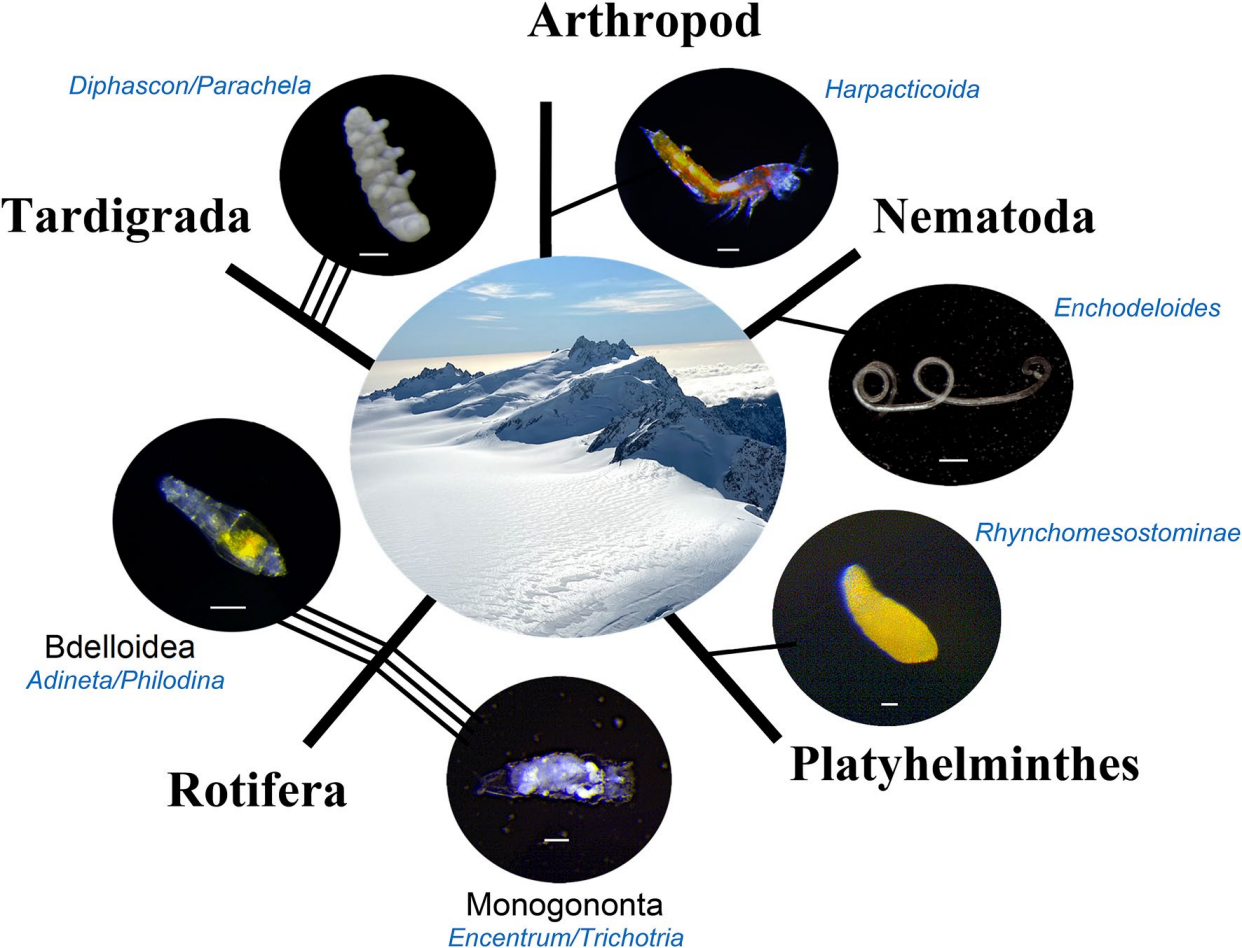
Animals in glacier ice collected from New Zealand’s Southern Alps. Species from five metazoan phlya are represented: Arthropoda, Nematoda, Platyhelminthes, Rotifera (with Classes Bdelloidea and Monogononta) and Tardigrada, collected from Fox, Franz Joseph and Whataroa Glaciers, respectively. At least 12 new species were identified, indicated by lines connected to respective images (e.g., three species of Tardigrada, etc.); genera designations were estimated by nuclear and mitochondrial barcoding in comparison with closest GenBank matches (see Table 1, Suppl. Info.). Central image shows the accumulation zone at the Franz Joseph Glacier collection site, 6,890 ft asl, just west of the continental ridge. Scale bars = 50 μm.

Pilot collections during mid-summer on Fox and Franz Joseph Glaciers (− 43.5319, 170.1268 and − 43.4902, 170.2408, respectively, Feb. 10, 2020) led to the identification of bdelloid Rotifera and Tardigrada populations. We returned in late autumn (April 28, 2020) following snowfalls totaling ~ 1 m, to collect ~ 80 L of surface ice from a single sampling location at three respective field sites along a NE  SW transect spanning ~ 35 km: Whataroa Glacier (− 43.4002, 170.5231; 4,859 ft), Franz Joseph Glacier (− 43.6575, 170.2374; 6,890 ft) and Fox Glacier (− 43.5331, 170.1271; 6,483 ft), all above the ELA (Fig. 1). At each field site, the upper snow layer (~ 1 m) was removed to expose ~ 1 m^2^ of hard surface ice, corresponding to that year’s weathered crust. The upper ~ 10 cm were chipped away, collected and processed for microinvertebrates accordingly. In total, > 5,000 individual, glacier animals were observed—mostly alive–in laboratory cultures, representing five animal phyla (Fig. 2; Table S1, Suppl. Info.): Arthropoda (Crustacea), Nematoda, Platyhelminthes, Rotifera (Bdelloid and Monogononta) and Tardigrada. Animal designations were based on morphology and closest alignments with deposited GenBank sequences, with the caveat that the global database is incomplete. Nonetheless, some Antarctic ancestries can be inferred (e.g., bdelloid rotifers, nematodes, tardigrades), with a likely mechanism of passive, global dispersal (e.g., windblown, avian)^28–32^.

Tardigrades were the dominant taxon across field sites, observed at densities between ~ 7–40 individuals/L; bdelloid rotifers and nematodes occurred at densities up to 3–4 individuals/L, while remaining taxa were less abundant (Table S2, Suppl. Info.). Additionally, an arachnid (Acari) and springtail (Collembola) were observed on the waters’ surface in laboratory cultures and likely reside on the glacial surface (Fig. S1, Suppl. Info.). All of the aforementioned animals were observed at the three respective field sites, respectively, suggesting that they comprise subpopulations along the southwestern coast, consistent with historical glacial dynamics and ice connectivity^33–35^.

**Figure 3:**
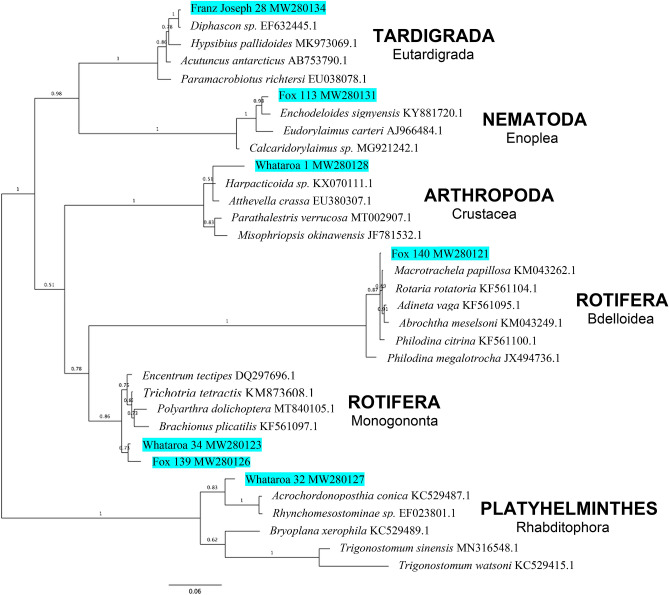
Midpoint-rooted Bayesian phylogeny across animal phyla based on 18S rRNA sequences. Blue highlighted taxa identify representative glacial specimens (designated by glacier followed by isolate number and GenBank accession) discovered in the current study. Related sequences with GenBank accession numbers appear in respective clades. Values along branches indicate node posterior probability (node support) and range from 0 to 1. Phylum and Class taxonomic designations are to the right.

Animal specimens were captured with a fine pipet, transferred individually and DNA barcoded using nuclear 18S ribosomal RNA (rRNA)^36^ and mitochondrial cytochrome *c* oxidase subunit 1 (CO1)^37^ primers. More than 90 individual specimens across the five animal phyla were processed identifying at least 12 putative species (Fig. 2; Fig. S2, Suppl. Info.), all of which appear new to science and, with the exception of bdelloid Rotifera^19^, not previously reported in glacier ice. Species boundaries were estimated using previously proposed thresholds of sequence divergence for nuclear and mitochondrial barcoding (e.g., ~ 10% divergence at CO1^38, 39^; 0.5–1% at 18S^40^) coupled with 18S rRNA Bayesian phylogeny across glacial phyla and related species (Fig. 3), collectively supporting the designation of discovered taxa as undescribed species (formal taxonomic descriptions of new taxa will be reported elsewhere).

**Figure 4:**
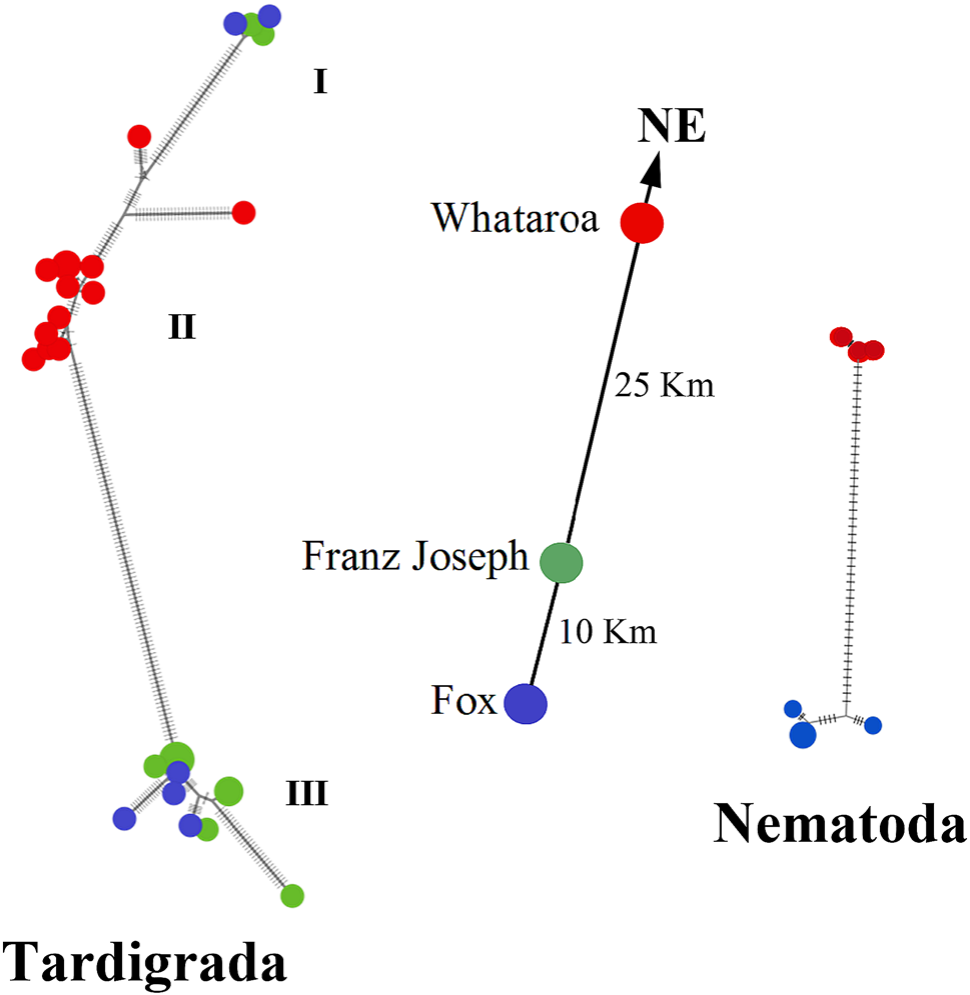
Haplotype networks depicting evolutionary relationships within Tardigrada and Nematoda populations. Each coloured circle represents a haplotype (i.e., a unique DNA sequence in a population) with radius proportional to number of individuals, collected along a NE transect at Fox, Franz Joseph and Whataroa Glaciers, accordingly (distance between glacier field sites indicated). The mitochondrial CO1 locus was successfully amplified in 29 tardigrades (with 25 haplotypes; GenBank accessions MW262004-MW262032) and eight nematodes (with six haplotypes; GenBank accessions MW262759-MW262766). DNA sequences were aligned in MEGA6^55^ and analyzed by HaplowebMaker^59^. Ticks along connector lines (edges) indicate mutational steps between individuals. Tardigrade clusters I and III represent separate species by delimitation criteria (~ 10% divergence at CO1 from cluster II; ~ 20% divergence between clusters I and III) that co-occur on Fox and Franz Joseph Glaciers, and appear to disperse actively between these two glaciers. Nematodes on Fox and Whataroa Glaciers, 35 km apart, displayed ~ 7% divergence at CO1, with no apparent gene flow between populations.

The onset of New Zealand glaciation occurred in the late Pliocene^41–43^. By applying a mitochondrial divergence rate of approximately 2% per million years for invertebrate taxa^15, 19, 44^, many species identified within respective phyla diverged prior to the onset of glaciation and arrived independently upon the onset of glaciation, while other putative species pairs (often found in sympatry) are more shallowly divergent and appear to have speciated thereafter. For instance, up to seven putative species of tardigrades are recognized using mitochondrial DNA divergence thresholds of 3%^45, 46^ (Fig. S2, Suppl. Info.), with divergence estimates that pre- and postdate glaciation. Note that such divergence thresholds (i.e., 3–10%) do not always estimate species diversity accurately and thus detailed taxonomic treatment of specimens is required to evaluate the extent of putative new species identified here. Representative haplotype networks for single- (Nematoda) and multispecies complexes (Tardigrada) (Fig. 4), suggest that glacier animals have and continue to disperse actively between coastal glaciers over geological time; moreover, mitochondrial DNA divergence patterns (Fig. 4) (i.e., exceeding previously proposed species boundary thresholds; Table S3, Suppl. Info.) support the persistence of these glacier animals throughout the Pleistocene.

The unexpected discovery of such animal diversity in New Zealand’s Southern Alps raises two important evolutionary questions. First, does this habitat represent an anomalous ecosystem that is driven by rainforest proximity and turbulent climatic winds, or does comparable animal diversity occur in glaciers worldwide? Limited data is available to assess this question, but to date North American and Icelandic glaciers appear restricted to monophylum animal representatives (Annelida and Rotifera, respectively) above the ELA^15, 19^. Secondly, the independent evolution of disparate animal phyla to the harsh and physiologically challenging conditions of glacial life above the ELA highlights the adaptive plasticity among microinvertebrate Animalia, raising the question of whether convergent mechanism(s) and/or novel biological strategies have facilitated their respective transitions into glacier ice. Previous studies show that glacier residents across domains of life^8, 47^, and particularly the North American glacier ice worm^8, 48, 49^, display enhanced purine anabolism that may compensate for cold temperature stress and lethargy^50–52^; this putative metabolic contribution to other glacial fauna remains an intriguing unknown, but now a testable hypothesis.

## Methods

### Specimen collection

Ice samples were taken from the top ~ 10 cm of glacier surfaces, chipped away and collected with EtOH-sterilized field equipment (shovels, picks) that were washed thoroughly between collections. Glacier ice was stored in 20 L plastic containers, transported to the University of Otago and thawed slowly at 4 °C over several days. To observe microinvertebrate specimens, melted glacier water was gravity filtered through Whatman 1 paper employing a Bückner funnel, viewed by stereomicroscopy and sorted into phylogroups based on morphology. Images were captured with a Leica M205 C stereomicroscope using LAS software.

### DNA extraction and PCR

Individual microinvertebrates were captured in 1–3 μl of glacier meltwater using a fine pipet and transferred into 7 μl of 70% EtOH for storage. To extract DNA, EtOH was evaporated on a 65 °C heat block for ~ 5 min with lid open, and 10 μl of a solution containing 25 mM Tris pH 8.5, 50 mM KCl, 5 mM MgCl_2_, Proteinase K (20 μg/μl) was added. Following incubation at 55 °C for 20 min, Proteinase K was inactivated by heating at 95 °C for 2 min and 1 μl was removed for polymerase chain reaction (PCR) analysis. DNA samples representing individual glacier specimens are archived in the laboratory of PKD. PCR reactions contained 1X Takara mix (Takara, Japan), 0.4 μM respective barcode primers [18S rRNA^36^; cytochrome *c* oxidase subunit 1 (CO1)^37^], 1 μl template in a total reaction volume of 25 μl balanced with H_2_O. Primers were: 18S2a-GATCCTTCCGCAGGTTCACC, 18S11b-GTCAGAGGTTCGAAGGCG^36^; HCO-TAAACTTCAGGGTGACCAAAAAATCA, LCO-GGTCAACAAATCATAAAGATATTGG^37^, respectively. Conditions for PCR were 95 °C for 2 min, then 94 °C (20 s)/45 °C for CO1, 54 °C for 18S rRNA (40 s)/72 °C (45 s) for 35 cycles, then 72 °C for 5 min. Aliquots were run on 0.8% agarose gels with EtBr and visualized by UV light. Positive samples were sequenced on both strands with respective PCR primers at the Genetics Services Facility (University of Otago, Dunedin).

### DNA sequence and data analyses

Sanger-sequenced DNA chromatograms were assembled and trimmed to remove primer sequence and low-quality base reads using 4 Peaks software^53^. BLASTn searches of assembled and cleaned sequences against the GenBank non-redundant nucleotide database were performed in 4 Peaks. New multi-sequence alignments were created within each phylum by combining new sequences with existing sequences drawn from Genbank (Suppl. Info., Table S1) using MAFFT v7.450^54^ employing default parameters. Using these alignments pairwise genetic distances were calculated by the Kimura 2-parameter correction in MEGA6^55^. MrBayes v3.2.7a^56^ was used to infer phylogenetic relationships among animal taxa by the General Time Reversible model of molecular evolution with invariant sites and a gamma distribution of rates (GTR + I + G). MrBayes was run on the CIPRES Science Gateway^57^ for 100 million Metropolis-coupled Markov Chain Monte Carlo (MCMCMC) generations with one cold and three heated chains, sampling every 10,000 generations. The R package RWTY v1.0.1^58^ was used to assess convergence of MCMCMC runs ensuring that the posterior sample was stationary and that the posterior sample of trees in independent runs recovered similar posterior probabilities for nodes. After evaluation, the first 50% of trees were removed as burn-in and the remaining sample was retained to infer a majority rule consensus tree. Haplotypes, as defined by unique CO1 sequences within a population, were created for each phylum by HaplowebMaker^59^ using default parameters (delimiter, mask error, radius proportion), and TCS software^60^, a Java program to estimate gene genealogies including multifurcations and/or reticulations by statistical parsimony^61^.

## Supplementary Information

Supplementary Information
