# Supplementary material for: Five animal phyla in glacier ice reveal unprecedented biodiversity in New Zealand's Southern Alps
Source: Sci Rep. 2021 Feb 16;11:3898. doi: 10.1038/s41598-021-83256-3 (PMC7887191; doi:10.1038/s41598-021-83256-3)
Supplement: Supplementary file 1 — Supplementary Information [file 41598_2021_83256_MOESM1_ESM.docx]

Five Animal Phyla in Glacier Ice Reveal Unprecedented Biodiversity in New Zealand's Southern Alps

^*1^Daniel H. Shain, ^2^Philip M. Novis, ^3^Andrew G. Cridge, ^4^Krzysztof Zawierucha, ^1^Anthony J. Geneva, ^3^Peter K. Dearden

^1^Biology Department, Rutgers The State University of New Jersey, Camden, New Jersey 08103 USA

^2^Allan Herbarium, Manaaki Whenua-Landcare Research, Lincoln 7608, New Zealand

^3^Genomics Aotearoa and Department of Biochemistry, University of Otago, Dunedin 9054, New Zealand

^4^Department of Animal Taxonomy and Ecology, Adam Mickiewicz University in Poznań, 61-614 Poznań, Poland

*Corresponding author

dshain@camden.rutgers.edu

**Supplementary Information**


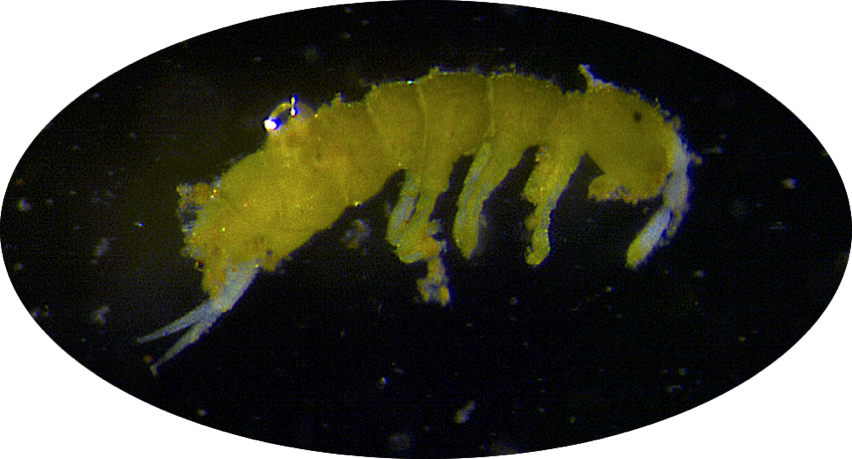

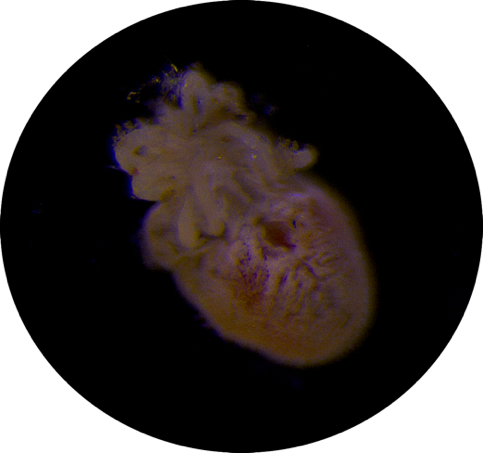


**Figure S1.** Glacier arthropods collected from New Zealand’s Southern Alps glaciers above the equilibrium line altitude (ELA). Based on DNA barcoding, the arachnid (Acari; left) is a species of *Nanochestes;* Collembola (right) is a species of *Folsomia*. Both were observed on the surface of laboratory water cultures and likely reside on the glacier surface. Scale bars = 200 μm.

**
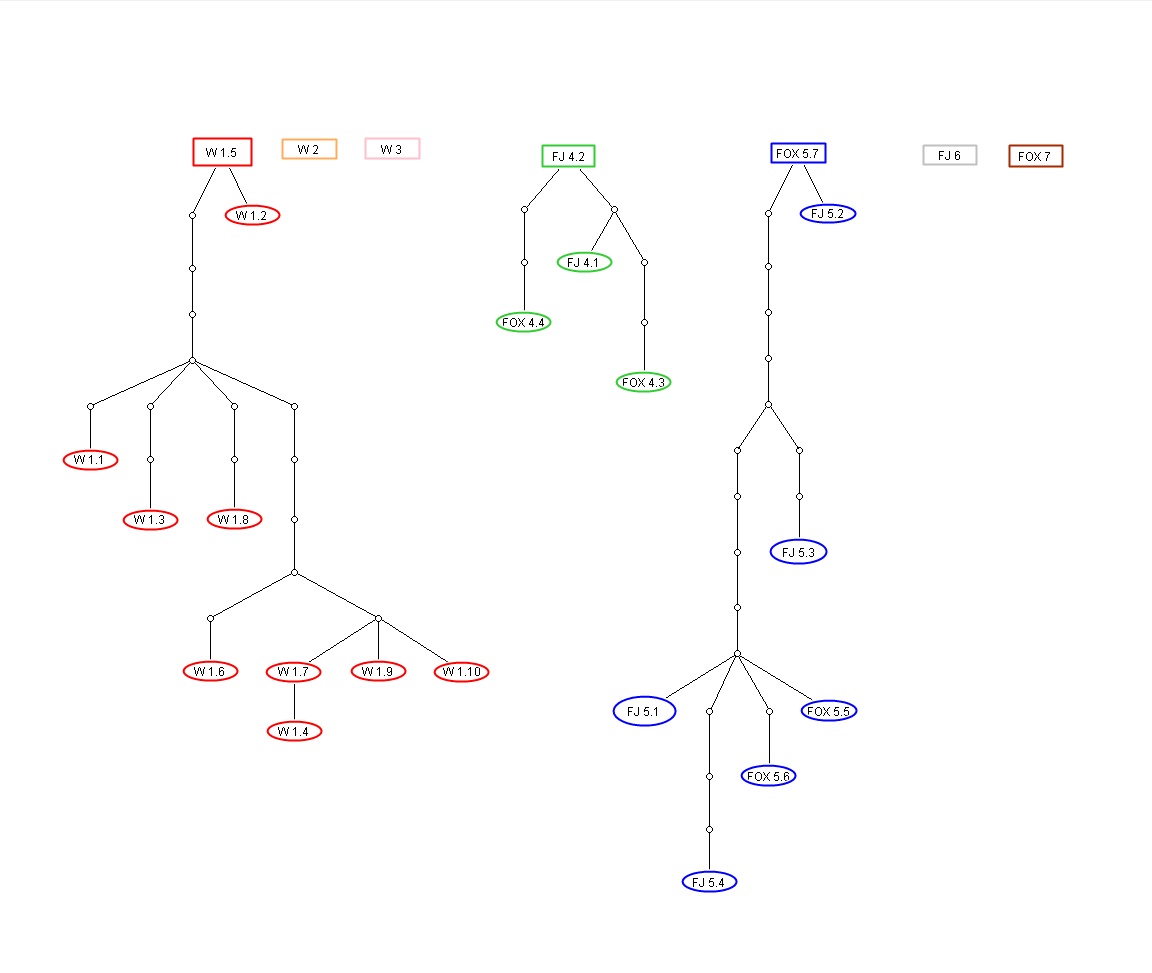
Figure S2A.** Tardigrade haplotype lineages prepared in TCS software^60,61^. Colored boxes represent putative tardigrade species (7 total) which exceed proposed species-level CO1 divergence thresholds of 3%^44,45^. Ovals identify individuals, nodes represent mutational steps between individuals. W - Whataroa Glacier, FJ - Franz Joseph Glacier, FOX - Fox Glacier.


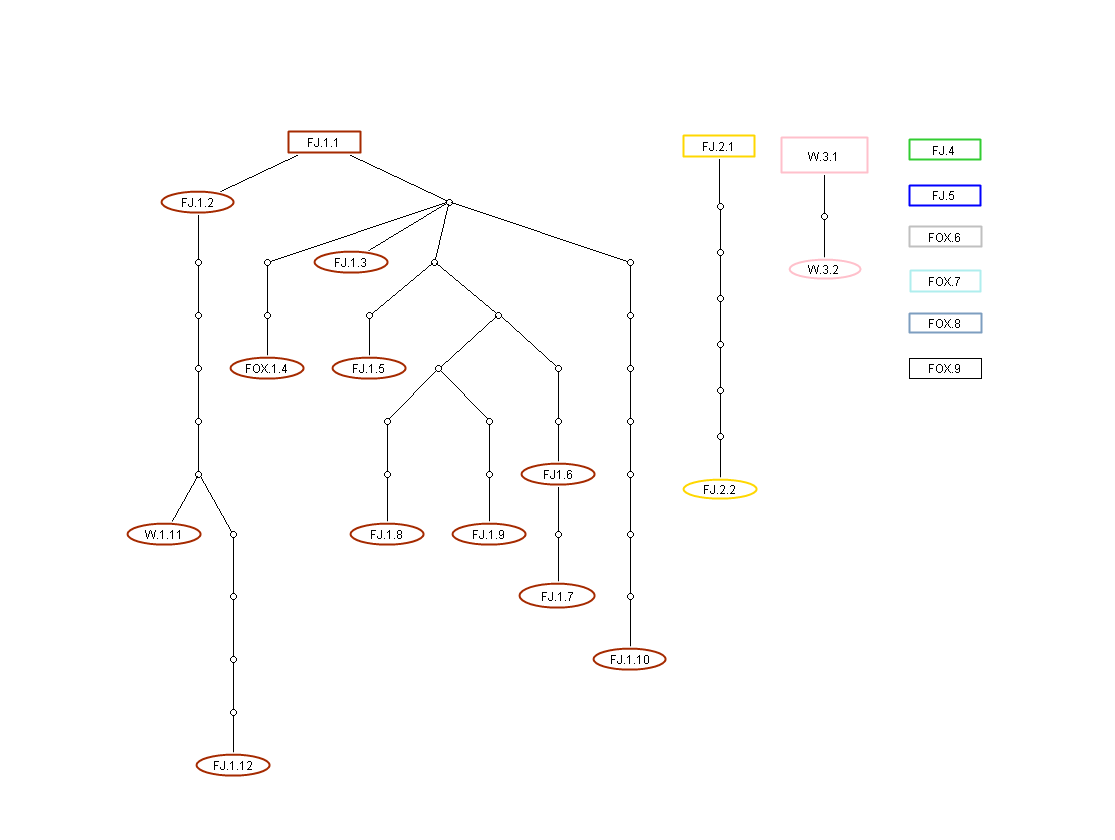


**Figure S2B.** Bdelloid Rotifera haplotype lineages (9 total).


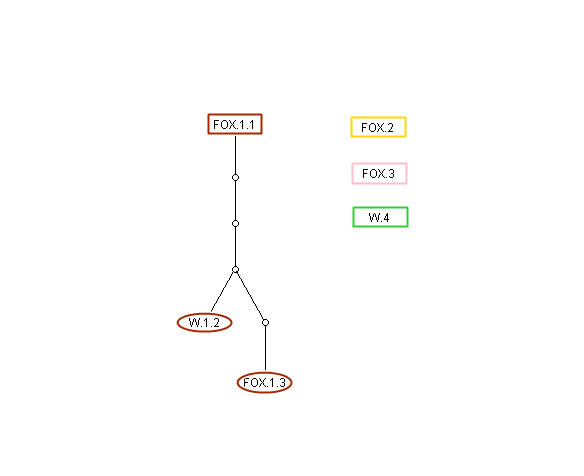


**Figure S2C.** Monogononta Rotifera haplotype lineages (4 total).


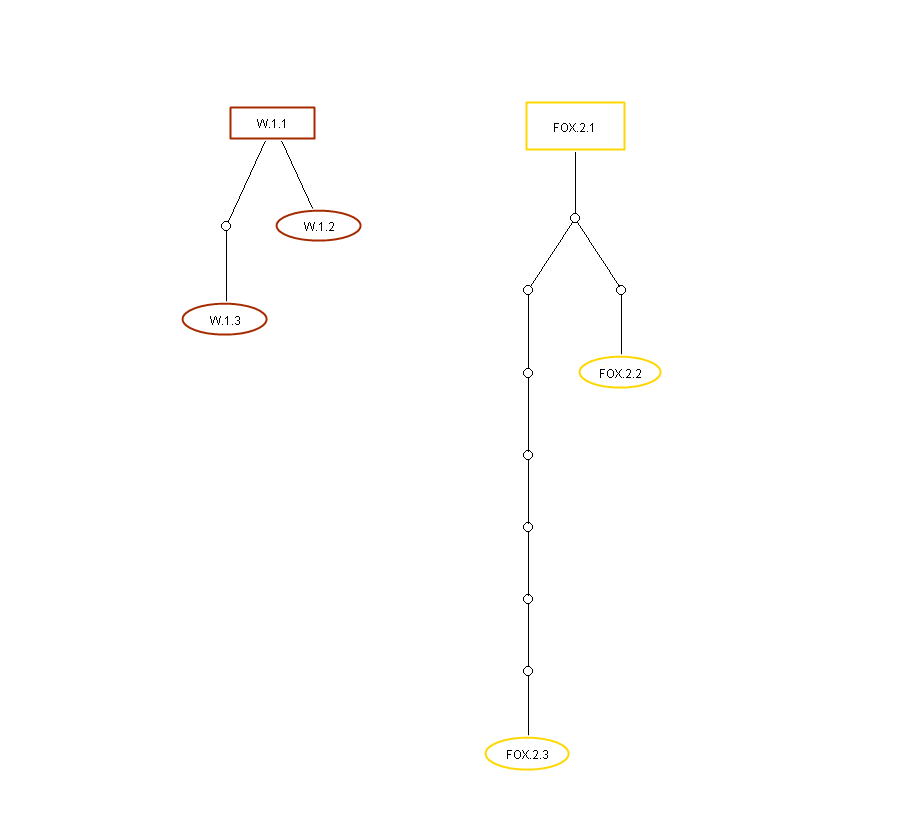


**Figure S2D.** Nematoda haplotype lineages (2 total).

**TaTable S1.** Collective data on representative animal sequences [18S rRNA and cytochrome *c* oxidase subunit 1 (CO1)] from Fox, Franz Joseph (FJ) and Whataroa (W) Glaciers, NZ.

| Phylum  (Class) | Indiv. | Locus | Accession number | Closest BLAST alignment | Identity | Geographic  region |
| --- | --- | --- | --- | --- | --- | --- |
| Arthropoda  (Arachnida) | W5 | 18S | MW280133 | *Nanochestes sp.*  (KY922107.1) | 80.6% | Caribbean |
| Arthropoda  (Arachnida) | W3 | CO1 | MW266571 | *Nanorchestes sp.*  (MF915553.1) | 78.2% | North America |
| Arthropoda  (Collembola) | W8 | 18S | MW259072 | *Folsomia penicula*  (JN981026.1) | 98.9% | Asia |
| Arthropoda  (Crustacea) | W1 | 18S | MW280128 | *Harpacticoida sp.*  (KX070111.1) | 96.8% | Marine, northeast Pacific |
| Arthropoda  (Crustacea) | W1 | CO1 | MW266572 | *Harpacticoida sp*.  (KP845479.1) | 80.1% | New Zealand |
| Nematoda | Fox113 | 18S | MW280131 | *Enchodeloides signyensis*  (KY881720.1) | 98.5% | King George Island, Antarctica |
| Nematoda | W66 | 18S | MW280132 | *Enchodeloides signyensis*  (KY881720.1) | 98.5% | King George Island, Antarctica |
| Nematoda | Fox113 | CO1 | MW262763 | *Dorylaimidae sp.*  KJ124469.1 | 83.3% | Antarctic Peninsula |
| Nematoda | W66 | CO1 | MW262761 | *Dorylaimidae sp.*  KJ124469.1 | 81% | Antarctic Peninsula |
| Platyhelminthes | W32 | 18S | MW280127 | *Rhynchomesostominae sp.* (EF023324.1) | 95.7% | North America |
| Rotifera  (Bdelloidea) | Fox140 | 18S | MW280121 | *Philodina sp.*  (MH429976.1) | 99.86% | Asia |
| Rotifera  (Bdelloidea) | W50 | 18S | MW280122 | *Philodina sp.*  (MH429976.1) | 99.86% | Asia |
| Rotifera  (Bdelloidea) | Fox140 | CO1 | MW266564 | *Bdelloidea sp.*  (KJ543624.1) | 81.5% | Antarctica Enderby Sector |
| Rotifera  (Bdelloidea) | W50 | CO1 | MW266544 | *Adineta sp.*  (KJ543628.1) | 86.8% | Antarctica Enderby Sector |
| Rotifera  (Bdelloidea) | FJ5 | CO1 | MW266549 | *Bdelloidea sp.*  (KJ543625.1) | 87.5% | Antarctica Enderby Sector |
| Rotifera  (Bdelloidea) | Fox136 | CO1 | MW266562 | *Bdelloidea sp.*  (KJ543623.1) | 84.1% | Antarctica Enderby Sector |
| Rotifera  (Bdelloidea) | Fox137 | CO1 | MW266563 | *Bdelloidea sp.*  (KJ543623.1) | 89.8% | Antarctica Enderby Sector |
| Rotifera  (Bdelloidea) | Fox121 | CO1 | MW266561 | *Macrotrachela jankoi* (MT180987.1) | 83.4% | Antarctica cryoconite |
| Rotifera  (Monogononta) | Fox139 | 18S | MW280126 | *Trichotria tetractis* (KM873608.1) | 97.6% | Cultured |
| Rotifera  (Monogononta) | W34 | 18S | MW280123 | *Encentrum tectipes* (DQ297696.1) | 98.7% | Cultured |
| Rotifera  (Monogononta) | Fox134 | CO1 | MW266567 | *Trichocerca sp.* (LC215604.1) | 75.2% | L. Kasumigaura, Japan |
| Rotifera  (Monogononta) | Fox135 | CO1 | MW266568 | *Lecane sp.*  (JX216676.1) | 75.2% | Rio Pedro Baranda, Mexico |
| Rotifera  (Monogononta) | W34 | CO1 | MW266565 | *Helina spinosa*  (KC499745.1) | 76% | Aleutian Islands, Alaska |
| Tardigrada | FJ28 | 18S | MW280134 | *Diphascon sp.* (EF632445.1) | 99.45% | S Orkney Islands Antarctica |
| Tardigrada | FJ28 | CO1 | MW262023 | *Diphascon puniceum* (KP013597.1) | 77.2% | S Orkney Islands Antarctica |
| Tardigrada | Fox7 | CO1 | MW262027 | *Parachela sp.,* (KJ856930.1) | 75.6% | Antarctica |
| Tardigrada | W42 | CO1 | MW262008 | *Parachela sp.,* (KJ856933.1) | 77% | Antarctica |
|  |  |  |  |  |  |  |

**Table S2.** Densities of glacier animals across field sites (individuals / L). For specimens at densities greater than ~1 / L, three representative 20 x 20 mm grids in filtered laboratory cultures were counted for individuals (dead or alive). Total animal estimates are based on extrapolations from sample aliquots. Numbers presented as the mean +/- SE (P<0.05).

| Animal | Fox Glacier | Franz Joseph  Glacier | Whataroa  Glacier | Total  Observed |
| --- | --- | --- | --- | --- |
| Tardigrada | 27 +/- 8.3 | 7.5 +/- 1.2 | 37.9 +/- 2.6 | ~4,000 |
| Rotifera (Bdelloidea) | 3 +/- 1.2 | <1 | <1 | ~500 |
| Nematode | 4.1 +/- 2.4 | <0.5 | <0.5 | ~300 |
| Platyhelminthes | <1 | <1 | <1 | ~100 |
| Rotifera (Monogononta) | <1 | <1 | <1 | ~100 |
| Crustacean | <0.5 | <0.5 | <1 | ~40 |
| Arachnida | <0.5 | <0.5 | <1 | ~50 |
| Collembola | <0.5 | <0.5 | <1 | ~80 |

Table S3. Kimura distance matrices comparing CO1 divergence (%) between individuals.
